# The Role of Lipids in the Regulation of Immune Responses

**DOI:** 10.3390/nu15183899

**Published:** 2023-09-07

**Authors:** Chelsea Garcia, Catherine J. Andersen, Christopher N. Blesso

**Affiliations:** Department of Nutritional Sciences, University of Connecticut, Storrs, CT 06269, USA; chelsea.garcia@uconn.edu (C.G.); catherine.andersen@uconn.edu (C.J.A.)

**Keywords:** lipids, sphingolipids, saturated fats, Western-type diet, unsaturated fatty acids, immune response

## Abstract

Lipid metabolism plays a major role in the regulation of the immune system. Exogenous (dietary and microbial-derived) and endogenous (non-microbial-derived) lipids play a direct role in regulating immune cell activation, differentiation and expansion, and inflammatory phenotypes. Understanding the complexities of lipid–immune interactions may have important implications for human health, as certain lipids or immune pathways may be beneficial in circumstances of acute infection yet detrimental in chronic inflammatory diseases. Further, there are key differences in the lipid effects between specific immune cell types and location (e.g., gut mucosal vs. systemic immune cells), suggesting that the immunomodulatory properties of lipids may be tissue-compartment-specific, although the direct effect of dietary lipids on the mucosal immune system warrants further investigation. Importantly, there is recent evidence to suggest that lipid–immune interactions are dependent on sex, metabolic status, and the gut microbiome in preclinical models. While the lipid–immune relationship has not been adequately established in/translated to humans, research is warranted to evaluate the differences in lipid–immune interactions across individuals and whether the optimization of lipid–immune interactions requires precision nutrition approaches to mitigate or manage disease. In this review, we discuss the mechanisms by which lipids regulate immune responses and the influence of dietary lipids on these processes, highlighting compelling areas for future research.

## 1. Introduction

Inflammation is a prominent underlying factor in the development and pathogenesis of many chronic diseases. Globally, chronic diseases are the most significant causes of death, totaling to 60% of all deaths [1]. In the United States, more than 125 million people have at least one chronic disease, while more than 20% of individuals have more than one chronic condition [1]. Of these conditions, cardiometabolic diseases such as type 2 diabetes (T2D) and cardiovascular disease are top contributors to chronic inflammatory disease mortality, with heart disease accounting for one out of every three deaths and T2D being the seventh leading cause of death in the United States [1]. With national healthcare spending estimated at USD 4.3 trillion, over 40% of total spending comes from 12% of the population with five or more chronic diseases [2]. Even more, patients without any chronic conditions only make up 10% of total health care spending [2]. Thus, chronic disease impacts a large portion of the population and contributes to 90% of the nation’s health care costs. Beyond the economic costs, living with chronic disease can have a significant negative impact on quality of life by affecting physical, psychological, and social functioning [3]. 

Many chronic diseases are characterized by dysregulated lipid and immune responses. Chronic inflammation is a hallmark feature of several diseases with increased immune cell proliferation, circulating immune cell recruitment and migration into tissues, and proinflammatory cytokine production [1]. Lipid metabolism is also altered during chronic diseases, which can include high serum concentrations of total cholesterol, triglycerides, and low-density lipoprotein cholesterol (LDL-C), with low high-density lipoprotein cholesterol (HDL-C) concentrations. Furthermore, endogenous and dietary lipids can alter the inflammation/immune response in chronic disease. Thus, inflammation and lipid metabolism are crucial when researching the development, progression, and treatment of chronic disease.

In this review article, we summarize (1) the process of immune activation to stimuli and the specific roles of cells within the innate and adaptive immune systems, and we compare and contrast the activation of gut mucosal vs. systemic immune responses; (2) the role of endogenous (non-microbial-derived) lipids in directly regulating immune cell activation, differentiation and expansion, and inflammatory phenotypes; (3) current knowledge regarding the immunomodulatory effects of dietary lipids; and (4) factors that may modify lipid–immune interactions.

## 2. Regulation of Intestinal Mucosal and Systemic Immune Systems

Cells and components of the immune system are present throughout the entire body in different tissues and organs. The immune system works systemically or locally, centralized to a specific tissue such as the mucosal immune system. An essential function of the immune system is to mediate the identification of molecules as endogenous, or “self”, vs. foreign, or “non-self”. Accordingly, the immune system is encoded to recognize molecules that are present in several pathogens but not present in mammals, and it can also recognize specific molecules that are presented on immune cells that can prompt an inflammatory response [1]. The recognition of foreign or pathogenic molecules prompts immune activation and a coordinated cellular response to eliminate the foreign entity, resolve inflammation, and promote tissue healing and homeostasis. The inability of the immune system to respond properly leads to adverse outcomes for acute injuries and infection, and this can be linked to several metabolic and chronic diseases [2]. In addition, failure of the immune system to distinguish between “self” and “non-self” molecules leads to immune dysfunction underlying the cause of autoimmune diseases [3]. Effective regulation and functioning of the immune system require coordinated responses by the innate and adaptive immune systems, as described below.

The innate immune system provides an immediate defense system that is passed down through genetics and is present at birth [3]. Physical barriers provide an initial barrier to pathogens in epithelial cells, which can include tight junctions, a mucus layer, and cilia [1]. The innate immune system is based on the recognition of damage-, microbe-, and pathogen-associated molecular patterns by pattern recognition receptors, such as Toll-like receptors (TLRs) [4]. Responses from the innate immune system are non-specific and often include the complement system and immune cells capable of phagocytosis [3]. These immune responders work to protect against pathogens but also play a role in tissue homeostasis and repair [3]. The complement system identifies pathogenic molecules and performs targeted lysis via the opsonization of pathogens and the use of the membrane attack complex [5].

Activated immune cells can secrete cytokines and chemokines that can attract other immune cells and lipid mediators to the site of inflammation [1]. The main cells in the innate immune system are myeloid cells, which include monocytes, macrophages, neutrophils, natural killer cells, and dendritic cells. Natural killer cells contain cytotoxic granules that can eliminate affected cells (i.e., tumor cells and virus-infected cells) [6,7]. Upon the recognition of microbial pathogens, macrophages and neutrophils can perform phagocytosis, while neutrophils can also degranulate and trap pathogens using DNA fibers and proteins [8]. Monocytes can also perform phagocytosis, secrete chemokines, and respond to sites of inflammation [9]. Dendritic cells, as well as macrophages, are the major antigen-presenting cells of the innate immune system that coordinate the activation of the adaptive immune system [10]. These innate immune cells can rapidly proliferate in response to inflammation, but then they lower towards basal levels once the pathogen is removed or inflammation is resolved [11].

The adaptive immune system develops over time and through exposure to different pathogens and foreign molecules. The innate immune system primes the body for an inflammatory response and activates the adaptive immune system [3]. The adaptive immune system differs from that of the innate through its specificity and capability of memory. The adaptive immune system can mount a highly specific response to a distinct pathogen and create memory cells to defend against that pathogen in the future [12]. Lymphocytes (B and T cells) are the major cells involved in facilitating adaptive immune responses, including antibody- and cell-mediated immune responses [12]. B cells play a role in responding to antigens by secreting antibodies called immunoglobulins (Igs) [12]. These antibodies can circulate throughout the body, binding to pathogens and inhibiting their ability to bind to receptors on host cells. Antigen-bound pathogens can also serve as tags for phagocytic cells of the innate immune system [12]. In contrast, T cells carry out cell-mediated responses, where activated T cells can either act directly with a foreign molecule processing and presenting antigens or produce cytokines that recruit macrophages to eliminate the pathogen [12,13].

Similar to the role of macrophages and dendritic cells in the innate immune system, B cells are the major antigen-presenting cells in the adaptive immune system. Together, antigen-presenting cells can phagocytose pathogens, process antigens, and present peptides on the cell surface with major histocompatibility complex (MHC) proteins to T or B cells for recognition [3]. B cells have B-cell receptors that can detect specific antigens and catalyze an immune response [11]. The two conventional T cells are cytotoxic (CD8^+^) T cells and T helper (CD4^+^) cells, which recognize antigens bound to MHC class I and II molecules on antigen-presenting cells, respectively [4]. Once T cells are activated, they can proliferate and secrete cytokines that promote the activation of macrophages and the differentiation of B cells into plasma cells that can produce antibodies [11]. Upon activation, naïve T cells can further differentiate into distinct T-cell subsets with unique functions and inflammatory phenotypes based on the types of cytokines and growth factors present in the tissue environment. For example, T helper 2 (Th2) cells carry out humoral responses, T helper 1 (Th1) cells activate macrophages, and T helper 17 (Th17) cells mainly secrete interleukin (IL)-17 to activate and recruit neutrophils [14]. Some of the activated T helper cells will also differentiate into memory cells that will initiate an immune response to that specific pathogen upon repeat exposure [11].

Cells of the innate and adaptive immune systems mediate immune surveillance by circulating in blood and taking up residence in lymphoid (spleen and lymph nodes) and non-lymphoid tissues. Non-lymphoid tissues often contain specialized tissue-specific immune cell types and structures that help to maintain tissue homeostasis and pathogen defense mechanisms [15]. Among these tissue types is gut-associated lymphoid tissue (GALT)—a leukocyte-rich tissue in the mucosa of the gastrointestinal (GI) tract. GALT contains immune cells in the epithelium and lamina propria tissue layers, including antigen-presenting cells and lymphocytes, and it also contains immune cells within Peyer’s patches [16]. Peyer’s patches are aggregated lymphoid follicles surrounded by epithelia that contain microfold (M) cells [17]. M cells can transport antigens and bacteria from the lumen to resident immune cells either creating a tolerance or systemic immune response [17]. Within Peyer’s patches reside B cells that produce IgA-producing plasma cells, which play a role in T-cell responses when activated [18,19]. IgA is the major immunoglobulin in the intestinal mucosa and plays a role in the movement and the neutralization of bacteria in the mucosa [20]. At the base of crypts are Paneth cells, innate immune cells that secrete antimicrobial peptides into the lumen in response to bacteria and pathogens to inhibit their functions [21]. Antimicrobial peptides, with some including α-defensins, regenerating islet-derived III, and lysozymes, can directly affect the microbiome present [22,23,24]. Lastly, innate lymphoid cells (ILCs) are the innate counterpart of T cells located in abundance on mucosal surfaces [25]. Activated type 3 ILCs (ILC3) within the epithelium produce IL-22 and IL-17, which promotes intestinal homeostasis and repair [26,27,28].

The inner mucus layer, epithelium, and lamina propria are all exposed to the intestinal microflora and immune cells, with their interactions playing a vital role in intestinal homeostasis [29]. The mucus layer is the first physical defense against bacteria [29]. Epithelial cells are another physical defense against bacteria but can also facilitate cytokine and chemokine signaling [29]. Lastly, the epithelium and lamina propria contain lymphocytes that produce interferon-gamma (IFN*γ*) and can quickly respond to inflammation [30]. Dendritic cells can process antigens and present them to immune cells within GALT [31]. They can also activate regulatory T cells and promote the differentiation of T helper cells into regulatory T cells [32,33]. Regulatory T cells suppress the immune response creating a tolerance to antigen exposures contributing to intestinal homeostasis [34]. Epithelial proinflammatory T helper cells, such as Th17 cells, play a role in eliminating pathogens and inducing inflammation [33]. The gut microbiome can additionally promote the differentiation of naïve T cells into Th17 cells that secrete IL-17 or regulatory T cells [35,36]. There is a delicate balance between proinflammatory T helper cells and regulatory T cells in maintaining intestinal homeostasis. Intestinal epithelial cells (IECs) also produce IL-17, which promotes the expression of other chemokines [37].

While cells of the innate and adaptive immune systems play an essential role in facilitating pathogen defenses and tissue homeostasis, an imbalance of anti- vs. proinflammatory mechanisms can result in immune-mediated dysfunction systemically and at the tissue level. Accordingly, greater numbers of proinflammatory innate and adaptive immune cell subtypes are observed in blood, and lymphoid and non-lymphoid tissues in conditions associated with chronic inflammation, including obesity and metabolic syndrome, cardiovascular disease, T2DM, and metabolic (dysfunction)-associated fatty liver disease [38,39]. Similarly, inflammation and dysfunction of GALT is observed in inflammatory disorders affecting the GI tract, including inflammatory bowel diseases (IBDs), such as Crohn’s disease and ulcerative colitis [40,41]. Thus, identifying the mechanisms by which immune balance can be restored is essential to maintaining pathogen defense while mitigating inflammatory disease complications.

## 3. Impact of Dietary and Endogenous Lipids on the Intestinal Mucosal and Systemic Immune Systems

Modulation of dietary and endogenous lipids may be an effective strategy to promote immune balance and mitigate chronic disease risk. Lipids can alter immune activity through the regulation of cell membrane fluidity, lipid raft formation, transcription factor activation, and gene expression, serving as precursors for pro- vs. anti-inflammatory eicosanoids. Dietary lipids can also impact immune cell phenotype, activation, proliferation, migration and infiltration, and cytokine production. As a result, exogenous (dietary and microbial-derived) and endogenous (non-microbial-derived) lipids play a direct role in regulating immune cell activation, differentiation and expansion, and inflammatory phenotypes. Accordingly, chronic diseases characterized by hyperlipidemia or altered lipid metabolism often exhibit an immune imbalance and inflammation. The following sections outline the role of lipids in regulating systemic and GALT-specific immune pathways within the context of chronic disease, with a specific focus on dietary lipids.

### 3.1. Saturated Fats

Diets high in saturated fat have been shown to increase inflammation and the risk of chronic inflammatory diseases. Saturated fatty acids, the major component of a high-fat diet (HFD), and their effects on the immune system have been extensively reviewed [42,43]. Mice fed a diet exclusively of saturated fatty acids for 2 weeks exhibited an increased inflammatory response to a systemic lipopolysaccharide (LPS) injection, which led to increased endotoxemia and its related mortality [44]. Palmitic acid, stearic acid, myristic acid, and lauric acid are some of the most common saturated fatty acids found in the diet. Macrophages treated with 100–500 μM of palmitic acid had increased monocyte chemoattractant protein 1 (Mcp1), Il6, IL8, and Cxcl10 expression compared to controls and secreted neutrophil-attracting nucleotides [45,46,47,48]. In contrast, myristic acid (100 μM) was only able to increase Mcp1 expression in treated macrophages [46]. Both myristate and palmitate reduced the phagocytic capacity of treated macrophages compared to that of controls, with phagocytic capacity correlating with the degree of fatty acid unsaturation [49]. Palmitic acid activates TLR4 on dendritic cells stimulating the production of IL-1*β* [50]. Diets high in saturated fats can also reduce the phagocytic capacity of peritoneal macrophages and natural killer cell activity in mice [51]. Candler et al. reviewed the effects of saturated fatty acids on lymphocyte proliferation and reported that they can have a modest effect on lymphocyte proliferation at times, but the effect is often not strong [52,53,54]. Interestingly, long-chain fatty acids can mitigate mesenteric lymphocyte proliferation [52]. Similar to macrophages, palmitic acid can induce the activation of T cells as indicated by increased inflammatory cytokine secretion, including tumor necrosis factor alpha (TNF*α*), IL-1*β*, IL-2, IL-6, IL-8, and IL-10 [55].

There are multiple lines of evidence demonstrating that palmitic acid, one of the most common saturated fatty acids, is a TLR4 ligand activating nuclear factor kappa B (NF-κB) and NLR family pyrin domain containing 3 (NLRP3) inflammatory pathways in macrophages, dendritic cells, and mast cells [50,56,57,58]. However, recent evidence suggests that saturated fatty acids do not directly activate TLR4 in macrophages but rather promote inflammation by altering macrophage lipidome and phenotype [59]. TLR4 dimerization or endocytosis does not occur in palmitate-treated macrophages; in addition, palmitate activation of JNK is not mitigated by pharmacological inhibition of TLR4 [59]. Instead, palmitate is able to alter the lipidome of macrophages with a decrease in phosphatidylcholines and an increase in phosphatidylethanolamines and ceramides that alter macrophage phenotype [59]. Lancaster et al. suggests that palmitate metabolism is critical for its inflammatory properties and can promote the M1 polarization of macrophages [59]. Other saturated fatty acids, including lauric acid and stearic acid, can also promote inflammation dependently and independently of TLR signaling [60,61].

**Figure 1:**
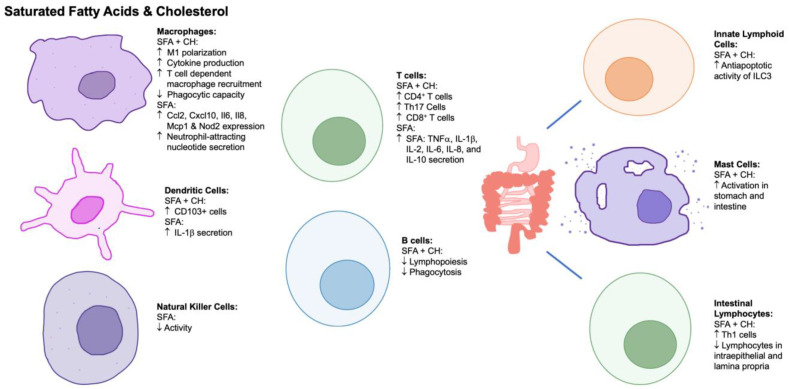
The effects of saturated fatty acids and cholesterol on innate, adaptive, and mucosal immune cells. Abbreviations: ↑, increase; ↓, decrease; Ccl2, chemokine ligand 2; CD4, cluster of differentiation 4; CD8, cluster of differentiation 8; CD103, cluster of differentiation 103; CH, cholesterol; Cxcl10, C-X-C motif chemokine ligand 10; IL, interleukin; ILC3, type 3 innate lymphoid cell; Mcp1, monocyte chemoattractant protein 1; Nod2, nucleotide-binding oligomerization domain containing 2; SFA, saturated fatty acids; Th1, T helper type 1 cells; Th17, T helper type 17 cells; TNF, tumor necrosis factor.

To review, the effects of saturated fatty acids impact many immune cells of the innate, adaptive, and tissue-specific immune systems (Figure 1). Regarding the innate immune system, the consumption of saturated fatty acids alters the phenotype/activity of macrophages, and there is an increase in M1 activation, cytokine production, and nucleotide release [45,46,47,48]. Further, saturated fats decrease macrophage phagocytic capacity and lower natural killer cell activity [49,62]. In adaptive immune cells, saturated fatty acids increase T-cell cytokine production [55], with limited information on B-cell function. Thus, the consumption of saturated fatty acids should be limited for those with chronic inflammatory diseases to mitigate a proinflammatory immune response.

### 3.2. Sterols

Similar to saturated fat, excess cholesterol may promote the activation of proinflammatory immune cells of both the innate and adaptive immune systems (Figure 1). Cholesterol can impact immune inflammation; the risk and progression of chronic, infectious, and autoimmune [63] diseases; and intestinal health. Plasma membrane cholesterol content is also important for inflammatory signaling by supporting the formation of the TLR4 complex in lipid rafts [64,65]. The consumption of excess dietary cholesterol affects immune cell function, infiltration, and migration into tissues. There is evidence that cholesterol consumption in vivo causes macrophage infiltration and accumulation in adipose tissue contributing to systemic inflammation [66,67]. Cholesterol also affects mast cell activation and subsequent foam cell formation. A high-cholesterol Western diet (1.25% cholesterol *w*/*w*) in mice promotes systemic mast cell activation, and mast cell activation has been shown to promote the uptake of LDL-C by macrophages [68,69]. In T cells, an increased membrane cholesterol content promotes an inflammatory response [70]. Although there are reviews that highlight the role of cholesterol metabolism in lymphocyte function [71,72,73], there is not much evidence regarding the impact of dietary cholesterol on lymphocyte functions and proliferation, highlighting an area for future research.

There is limited evidence showing that dietary cholesterol is able to influence the intestinal immune system. When consumed in excess, cholesterol can increase IL-1β levels in the small intestine of mice 12 h after a gavage of oil and cholesterol, and it can worsen intestinal motility in zebrafish fed a high-cholesterol diet for 10 days [74]. It can also lead to increased levels of oxysterols, which can also modulate intestinal health [75,76]. In a diet-induced obesity model, a Western-type diet high in cholesterol caused defects in Paneth cell function via microbiome-mediated deoxycholic acid synthesis, which increased farnesoid X receptor (FXR) and type I IFN signaling after 4 weeks of feeding [77]. Many researchers use Western-type diets to investigate the effects of dietary cholesterol; however, these effects cannot be solely attributed to cholesterol content due to the increased fat and carbohydrates in these diets. Consequently, researchers need to identify the role of cholesterol under low- and high-fat conditions in immune cell function, especially in mucosal immune cells.

Phytosterols, including campesterol, stigmasterol, and sitosterol, are cholesterol-like compounds with a steroid skeleton found in plants, fruits, and vegetables [78]. Phytosterols have been shown to have pleiotropic effects with hypolipidemic, antioxidant, and anti-inflammatory properties among others [79]. However, due to the low bioavailability of phytosterols (0.5–2%) [80], these effects are likely due to indirect mechanisms. In humans, plant sterol supplementation from doses of 1.7 g/day to 2.5 g/day for 28 days—16 weeks did not alter serum inflammatory markers, TNF*α*, IL-6, or MCP-1, in adults with hypercholesterolemia and metabolic syndrome [81,82,83]. To test the effects of plant sterols on the inflammatory response in preclinical models, researchers isolated splenocytes from mice fed a mixture of plant sterols. Splenocytes isolated from apoE^−/−^ mice fed 2% soybean-derived sterols for 14 weeks and immunized with ovalbumin (mainly Th2-mediated) exhibited increased lymphocyte proliferation and mitigated inflammatory responses after stimulation with LPS compared to controls [84]. T-cell-dependent cytokine production and increases in IL-10 production were also seen in splenocytes isolated from these mice cultured with ovalbumin compared to controls [84]. However, spleen lymphocytes isolated from apoE^−/−^ mice fed 2% phytosterols for 48 *h* injected with or without turpentine did not affect proliferation [85]. Spleen lymphocytes isolated from apoE^−/−^ mice fed the same diet for 4 weeks treated with turpentine and concanavalin A increased IL-2 cytokine production [85]. Lastly, the Th1/Th2 ratio was increased in both apoE^−/−^ mice and C57Bl/6J mice after the consumption of the phytosterol diet and turpentine-induced inflammation (mainly Th1-mediated) compared to that of controls [85]. Thus, phytosterol consumption does not seem to affect circulating inflammatory cells in non-healthy clinical populations, while it can alter immune cell proliferation and cytokine secretion in ex vivo models.

### 3.3. Western-Type Diets

Western-type diets also commonly referred to as HFDs contain high proportions of saturated fatty acids, sugar, and cholesterol. The consumption of a HFD can induce chronic low-grade inflammation in central and peripheral tissues in mice [86]. A HFD can promote inflammation by increasing the pathogenicity of the gut microbiota, which can lead to increased gut permeability, systemic endotoxemia, and the activation of TLRs [87,88,89]. The consumption of a HFD also affects the innate immune system by increasing nucleotide-binding oligomerization domain containing 2 (NOD2) expression, another intracellular pattern recognition receptor, which induces NF-κB inflammatory signaling [90]. The increase in fatty acids from the diet and exposure to bacterial LPS can also induce intestinal cell secretion of IL-1β, IL-6, and TNF*α* [91,92,93,94]. This inflammatory state and the increased availability of lipids promote the M1 macrophage phenotype, further increasing inflammation via cytokine production, even after short-term feeding [87,89,95]. Early on, the consumption of a high-saturated and high-cholesterol diet can also promote the early circulation of foamy monocytes that contribute to other inflammatory diseases such as atherosclerosis [96,97]. HFD consumption can also induce inflammation by increasing systemic levels of MCP-1 in Wistar rats [98]. Long-term HFD consumption causes an influx of macrophages into liver and adipose tissues, therefore reducing circulating monocyte levels [99,100]. Interestingly, ILC3 has anti-apoptotic activity under HFD conditions in the liver. HFD consumption for 12 weeks stimulates the production of IL-23 by M1 macrophages to promote the proliferation of ILC3, which produces and secretes IL-22, a cytokine that inhibits palmitate-induced apoptosis [101].

The consumption of a HFD also affects immune cells of the adaptive immune system. There is an increase in Th17 cells and CD103+ cells, a subset of dendritic cells, and immune cell infiltration of adipose tissue in mice fed a HFD [62]. However, recent evidence suggests that a HFD for 16 weeks can promote T-cell-dependent macrophage recruitment and helper T-cell and cytotoxic T-cell proliferation [102]. Even short-term HFD feeding (6 weeks) increased Th2 cytokines IL-10 and IL-13 in C57Bl/6J mice [103]. Interestingly, dietary fatty acids can alter the fatty acid composition of lymphocyte membranes and secretions, which can be deleterious to their function in excess [54,104,105]. For example, T cells isolated from the bone marrow of mice fed a HFD for 20 weeks had an increased secretion of palmitic and myristic acids [104]. Lastly, HFD can also suppress B-cell lymphopoiesis and impair B-cell phagocytosis after 18 and 16 weeks, respectively [106,107]. However, B-cell accumulation in visceral adipose tissue is associated with HFD-induced insulin resistance after 16 weeks [106].

There is evidence that a HFD can also affect GALT and mucosal immune homeostasis. The consumption of a HFD can decrease the protective intestinal mucosal layer [108,109,110] and increase inflammatory cytokine expression and the numbers of Th1 cells in the colon [98,111,112]. HFD feeding has also been shown to affect mast cells by increasing their activation and degranulation in the stomach and intestine [113,114]. Type I ILCs (ILC1) secrete IFN*γ* and TNF*α* in response to intracellular bacteria [28]. During dysbiosis, which can be caused by a HFD, there is an overabundance of ILC3- and ILC1-secreted Th1 and Th17 cytokines [28]. In addition, HFD consumption in mice alters the morphological appearance of GALT and induces atrophy of the intestine after 3 weeks [115]. The consumption of a HFD impairs the mucosal barrier by reducing goblet cells and mucin 2 (Muc2) expression after 11 weeks and 22 weeks in mice [108]. There is also a reduction in intraepithelial and lamina propria lymphocytes, even after one day of HFD feeding [115]. Tanaka et al. concluded that the availability of dietary free fatty acids (FFAs) in the intestinal lumen mediates the proinflammatory properties of HFDs in the intestinal immune system, as they found that statin treatment did not reverse the effects of a HFD [115].

Regarding the innate immune system, the consumption of a HFD alters the phenotype/activity of macrophages and dendritic cells. Consequently, there is an increase in macrophage M1 activation, cytokine production, and T-cell-dependent recruitment [87,89,95,102]. Lastly, HFDs impact adaptive immune cells and the mucosal immune system increasing Th2 cytokine production [103]. Increased fat consumption increases the anti-apoptotic activity of ILC3s, augments mast cell activation in the stomach and intestine, and can increase intestinal Th1 cells but decrease intraepithelial lymphocytes and lymphocytes within the lamina propria [87,101,111,112,113,114,115]. Overall, the effects of Western-type diets impact many immune cells of the innate, adaptive, and tissue-specific immune systems. Thus, the consumption of a Western-type diet should be limited for those with chronic inflammatory diseases to mitigate immune cell activation and cytokine production. However, these effects may not be solely due to the high lipid content of the diet. While many of the effects of Western-type diets are attributed to the saturated fat content, there are also higher sucrose and fructose contents that may have their own confounding effects [116,117,118]. 

### 3.4. Unsaturated Fatty Acids

Supplementation of dietary unsaturated fatty acids has been commonly used as a potential preventative and therapeutic measure in preclinical disease models using HFDs. Dietary unsaturated fatty acids can include omega-3, omega-6, and omega-9 fatty acids. Omega-3 fatty acids include eicosapentaenoic acid (EPA), docosahexaenoic acid (DHA), and alpha-linolenic acid, while the most common omega-9 fatty acid is oleic acid. Omega-6 fatty acids include linoleic acid, gamma linoleic acid, and arachidonic acid, while the oxidation of arachidonic acid and other polyunsaturated fatty acids can yield eicosanoids. Eicosanoids can include prostaglandins, leukotrienes, and lipoxins that can either promote or resolve inflammation. These fatty acids can have either anti-inflammatory or proinflammatory effects based on the type, concentration, and exposure.

Dietary fatty acids, such as omega-3, omega-6, and omega-9 fatty acids, can influence the production of cytokines, such as IL-1, IL-2, and TNF*α* [119]. Many preclinical and clinical studies show that *n*-3 supplementation can mitigate the production of inflammatory cytokines [120,121,122]. Thp-1 macrophages treated with 50 μM of EPA, DHA, or EPA + DHA reduced the expression of Il1*β*, chemokine ligand 2 (Ccl2), and Tnf after 24 h of treatment [120]. In human endothelial cells, 10 μM of DHA treatment for 96 *h* mitigated the IL-1*α* induction of IL-6 and IL-8 production and secretion [123]. Interestingly, in male C57BL/6 mice supplemented with long-chain *n*-3 polyunsaturated fatty acids (PUFAs) in the form of phospholipids or in the form of triglycerides for 8 weeks, serum MCP-1 and IL-6 concentrations were reduced along with their gene expression in epididymal adipose tissue [124]. Omega-9 fatty acids from olive oil were able to increase IL-2 cytokine production in splenic lymphocytes isolated from mice fed 15% or 20% omega-9 fatty acids for 90 days and 8 weeks, respectively [125,126]. Lastly, omega-6 fatty acids can reduce TNF*α* production in rats but increase IL-2 production in mice [126,127]. Although the exact mechanisms are unknown, *n*-3 PUFAs can inhibit the expression of the CD25 alpha chain in the IL-2 receptor [128]. Omega-3 fatty acids are also reported to alter cytokine expression by modifying NF-κB activity via its dimer subunit composition [129]. Thus, reducing cytokine production can reduce inflammation by reducing the expression of genes associated with inflammatory immune cell expansion and recruitment.

Omega-3 fatty acids, EPA and DHA, have been shown to modify inflammation by altering cytokine production and monocyte phenotype. EPA and DHA treatment of human umbilical vein endothelial cells (HUVECs) at a concentration of 10 μmol/L markedly reduced monocyte adhesion via the suppression of platelet-activating factor synthesis after 6 h [130]. Similarly, monocytes isolated from healthy humans treated daily with 1.02 g of EPA and 0.69 g of DHA for 12 weeks impaired the ability of monocytes to stimulate HUVECs to recruit neutrophils [131]. In contrast, monocytes from hypertriglyceridemic patients in a crossover trial treated with 4 g/day of EPA and EPA + DHA for 14 days improved the monocyte phenotype by reducing CD11c levels on classical and intermediate monocytes, more so with the combination of both fatty acids [132]. In an obesity-associated tumor model, omega-3 fatty acids induced the apoptosis of protumor macrophages to mitigate tumor growth without affecting cytokine production [133]. DHA can also improve macrophage polarization in vitro by promoting M2 polarization dependent on peroxisome proliferator-activated receptor γ (PPARγ) signaling [134]. Interestingly, oleic acid, EPA, and DHA can increase the phagocytosis of unopsonized zymosan particles via murine macrophages [49,134]. Patients with rheumatoid arthritis were supplemented with either low- (27 mg/kg; 18 mg/kg) or high-dose (54 mg/kg and 36 mg/kg) EPA and a DHA dose or olive oil (6.84 g/day) for 24 weeks [135]. Those receiving high-dose EPA and DHA had reductions in serum IL-1 release and production, while those receiving olive oil saw nonsignificant reductions in IL-1 and increases IL-2 [135]. Omega-3 fatty acids are also efficient in mitigating inflammation in young adults and even more efficient in older women fed 2.4 g/day for 3 months via reductions in inflammatory cytokines, IL-1*β*, TNF*α*, and IL-6 [122]. Reductions in inflammatory cytokines and an improved monocyte phenotype can protect against inflammatory and vascular diseases such as cardiovascular disease [136].

PUFAs, like EPA and DHA, have been shown to inhibit cell division more strongly than saturated fatty acids, which may be due to alterations in membrane fluidity, cell cycle regulation, and the activation of transcription factors [129,137,138,139]. Lymphocytes from mice fed oleic-acid-rich olive oil showed a reduction in lymphocyte proliferation, but not as strongly as EPA and DHA compared to sunflower oil, which is rich in oleic and linoleic acids, and coconut oil, a major source of lauric acid [125]. However, it was found that DHA is not the driving anti-inflammatory contributor to fish oil intake in healthy men [140]. Kelley et al. found that 6 g/day of DHA for 90 days did not alter circulating IL-2-producing T cells, the circulating helper: suppressor T-cell ratio, circulating peripheral lymphocyte counts, or the serum IL-2 receptor [140]. Interestingly, dietary fatty acids can manipulate lymphocyte activity differently based on fatty acid concentrations [125,141,142]. For example, palmitoleic acid is only toxic to lymphocytes above 50 μM [143]. In addition, oleic acid and linoleic acid suppress proliferation at 75 and 100 μM yet promote expansion at 25 μM [144]. Lastly, dietary fatty acids can affect natural killer cell activity and the activity of phagocytic cells. Fish oil and olive oil consumption in preclinical studies result in the inhibition of natural killer cell activity compared to omega-6 fatty acids and saturated fatty acids [125,141,145]. Preliminary evidence in humans suggests that DHA and EPA can mitigate natural killer cell counts, but more research needs to be conducted to determine what factors alter the effects of DHA and EPA on natural killer cells, as there has been some contradicting evidence [146,147,148,149]. An increased proliferation of immune cells is a common characteristic of many diseases; thus, dietary approaches to decreasing their expansion could be therapeutic.

Studies evaluating the effects of fatty acids on the intestinal mucosal immune system are limited. A meta-analysis on the effect of fatty acids and IBD revealed a reduced risk of IBD with increased *n*-3 fatty acid consumption and attenuated colon inflammation [150]. In senescence-accelerated mice with spontaneous ileal inflammation, omega-3 fatty acids (8% *w*/*w*) for 16 weeks reduced macrophage infiltration and inflammatory cytokine expression mitigating intestinal inflammation [151]. In middle-aged rats, omega-3-rich fish oil impaired the mucosal barrier via decreased goblet cell counts and Muc2 expression [152]. In another study, rats were immunized with keyhole limpet hemocyanin via an intra Peyer’s Patches injection to identify the effect of fatty acids on immune cell responses [153]. Lymphocytes isolated from the thoracic duct displayed a higher proliferative response to mitogens when rats were fed for 6 weeks with a 20% perilla seed oil diet high in PUFA compared to a high-saturated-fat diet [153]. There has been no research conducted to identify the effect of PUFAs on immune cells of the mucosal immune system, including M cells and Paneth cells. Thus, there needs to be more research on the role of dietary fatty acids in the intestinal mucosal immune system in different disease models and in specific cell types.

**Figure 2:**
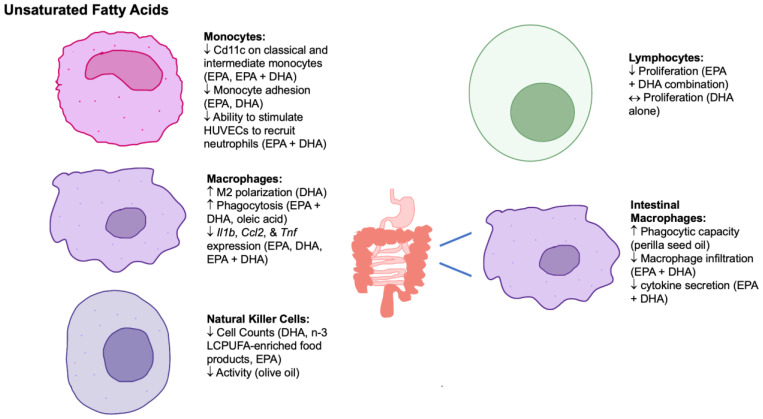
The effects of unsaturated fatty acids on innate, adaptive, and mucosal immune cells. Abbreviations: ↑, increase; ↔, no change; ↓, decrease; Ccl2, chemokine ligand 2; DHA, docosahexaenoic acid; EPA, eicosapentaenoic acid; HUVECs, human umbilical vein endothelial cells; Il1b, interleukin1b; LCPUFA, long-chain polyunsaturated fatty acid; Tnf, tumor necrosis factor.

To summarize, unsaturated fatty acids tend to have a beneficial effect on immune cells of the innate and adaptive immune systems (Figure 2). DHA can increase macrophage M2 polarization, decrease macrophage proinflammatory gene expression, and decrease monocyte adhesion and natural killer cell counts but do not alter lymphocyte proliferation [120,130,134,146]. In contrast, EPA has also been shown to decrease macrophage proinflammatory gene expression, monocyte adhesion, and natural killer cell counts but can also decrease Cd11c on classical and intermediate monocytes [120,130,132,147,148,149,154]. However, when in combination, EPA + DHA can increase macrophage phagocytic capacity; decrease Cd11c on classical and intermediate monocytes, macrophage proinflammatory gene expression, the monocyte ability to stimulate HUVECS to recruit neutrophils, and lymphocyte proliferation; and reduce intestinal macrophage infiltration and cytokine secretion [53,120,125,126,131,132,135,141]. Lastly, there is minimal evidence on the effect of other unsaturated fatty acids and oils rich in unsaturated fatty acids on immune cells. Oleic acid, the major fatty acid in olive oil, has been shown to increase the phagocytic capacity of macrophages, while olive oil consumption has been shown to decrease natural killer cell activity and lymphocytes [125,141,145]. Perilla seed oil is rich in alpha linolenic acid and has been shown to increase the proliferation of thoracic duct lymphocytes [153]. Consequently, unsaturated fatty acids contribute to the resolution of inflammation and may provide beneficial outcomes for those with inflammatory diseases.

### 3.5. Eicosanoids

As mentioned previously, eicosanoids, including prostaglandins and leukotrienes, can be derived from polyunsaturated fatty acids. Prostaglandins are produced from arachidonic acid via cyclooxygenases and prostaglandin synthases. Prostaglandin E_2_ (PGE_2_) is produced in the submucosa and prevents innate immune cells from responding to antigens via EP4 receptor activation [155]. EP4 signaling has been shown to protect against colitis and mucosal damage, helping to maintain gut homeostasis and epithelial proliferation while also limiting immune responses [155]. PGE_2_ has anti-inflammatory properties by influencing macrophages, ILCs, and epithelial cells [155,156,157,158], and it can also promote an anti-inflammatory neutrophil phenotype. For example, PGE_2_ can downregulate the production of TNFα and upregulate IL10 in macrophages [159]. Similarly, PGE_2_ suppresses B-cell proliferation via EP4 receptor activation and promotes the production of IgE antibodies by B cells [160]. However, PGE_2_ can also exacerbate intestinal inflammation by inhibiting mucosal regulatory T cells with intact gut microbiota in mice [161], and it can promote colitis in mice via increased dendritic cell IL-23 secretion contributing to inflammatory Th17 cells [162].

Prostaglandins were described above as mainly having anti-inflammatory effects against colon inflammation, yet prostaglandins are more commonly known for their proinflammatory role in acute inflammation. Prostaglandin synthesis is increased at sites of inflammation and plays a pivotal role in this process by regulating blood flow and pain sensitization [163,164]. Prostaglandins can increase arterial dilation, which increases blood flow to the site of inflammation, and can increase pain via peripheral sensory neuronal stimulation [165]. However, during chronic diseases, such as autoimmune disease and IBD, PGE_2_ can inhibit inflammatory cytokines via the suppression of NF-κB and promote intestinal health, as mentioned previously [166]. It seems that the inflammatory properties of prostaglandins are dependent upon the prostaglandin type itself and the concentration. Short-term acute exposure to PGE_2_ can induce acute inflammation, while chronic intermediate exposure to PGE_2_ attenuates chronic inflammation [166]. Cytokines can also promote a proinflammatory PGE_2_ phenotype [166]. Interestingly, only slightly elevated PGE_2_ levels play a role in inflammation resolution [166]. Further research is needed on lipids that have both pro- and anti-inflammatory properties like prostaglandins.

While some eicosanoids can promote inflammation, others can mediate inflammation, and these lipids are called specialized pro-resolving lipid mediators (SPMs) [167]. Some SPMs include resolvins, lipoxins, and protectins that can switch the inflammatory status of a cell by promoting the production of other SPMs and inhibiting the production of proinflammatory prostaglandins and leukotrienes [168]. Interestingly, SPMs can exert an anti-inflammatory effect without suppressing the immune response and maintaining homeostasis [169]. SPMs can also affect the activity of dendritic cells, T cells, and B cells [169]. The ability of dendritic cells to migrate, produce cytokines, and fully mature is influenced by SPMs, which reduce inflammation and antigen presentation [169]. SPMs work to inhibit the production of cytokines from T cells that can activate other T cells, B cells, and dendritic cells [169]. SPMs also promote the differentiation of T cells into regulatory T cells to suppress inflammatory cytokines while inhibiting the proliferation of Th1, Th2, Th17, and cytotoxic T cells [169]. Lastly, SPMs affect B cells by increasing antibody production; inhibiting IL-6 and IL-10 cytokine production; and promoting the production of innate response activator B cells that can activate dendritic cells, neutrophils, and monocyte production [169,170].

### 3.6. Sphingolipids

Sphingolipids are another class of lipid that can be found in the human diet and play a major role in regulating the immune system and intestinal immune system. Several reviews have highlighted the anti-inflammatory effects of dietary sphingomyelin [171,172,173,174]. The consumption of sphingomyelin, a phosphosphingolipid mainly found in animal products like milk and eggs, has been shown to have limited effects on serum, lymphatic, and tissue sphingolipid concentrations, with no change or modest reductions seen in preclinical and clinical models [175,176,177,178]. Regardless, many preclinical studies have demonstrated the anti-inflammatory effects of dietary sphingomyelin under inflammatory conditions [179,180].

The consumption of dietary milk sphingomyelin (0.1% *w*/*w*) for 10 weeks in a mouse model of diet-induced obesity attenuated systemic inflammatory markers [179,180]. Many systemic effects of dietary sphingolipids may occur through indirect effects, secondary to their inhibition of dietary cholesterol and fat [174]. However, it is possible that dietary sphingolipids may also directly affect the immune cells residing in the intestine. Milk sphingomyelin mitigated LPS-induced inflammation in RAW264.7 macrophages via its metabolites, as the inhibition of acid sphingomyelinase reversed the effects of sphingomyelin treatment [179]. Further treatment of macrophages with sphingomyelin metabolites, including ceramides and sphingosine, found that long-chain (C16) and very-long-chain (C24) ceramides, and sphingosine can inhibit the LPS activation of macrophages via *Tnf* and *Ccl2* expression without affecting cell viability or apoptosis [179]. However, dihydroceramides did not affect inflammation, cell viability, or apoptotic markers [179]. Further studies should be conducted to identify the effects of sphingolipids and their metabolites on other immune cells.

Host glycosphingolipids are targets of pathogen and microbial binding; thus, microbial–lipid interactions are important in inflammation [181]. Sphingosine, phytosphingosine, and sphinganine have reported bactericidal effects against *Streptococcus* infection [182]. Sphingomyelin also plays an essential role in the translocation of proteins required for clathrin-independent endocytosis [183]. Lipid rafts abundant in sphingolipids enhance bacterial phagocytosis [184]. Sphingolipids derived from the milk fat globule membrane demonstrate anti-bacterial properties against several in vitro infectious bacteria [185]. In contrast, pathogens can use host sphingolipids in lipid rafts to elude detection by immune cells [186]. C16-ceramide-abundant lipid rafts can increase proinflammatory cytokine expression and cell signaling, mediate CD40 clustering, and increase susceptibility to apoptosis [187,188,189,190].

Sphingolipids can modify the function of innate immune cells, including monocytes, dendritic cells, and mast cells. Mast cell activation is inhibited in response to exogenous ceramides and sphingomyelin, yet sphingosine-1-phosphate (S1P) can promote the activation of these cells and their actions [191,192,193,194]. In monocytes, ceramide-enriched LDL promotes the inflammatory and apoptotic effects of LDL, while S1P can have anti-apoptotic effects [195]. Monocyte cytokine release is augmented in response to sphingosine-loaded LDL particles, which enhance inflammation [195]. In addition, dendritic cell apoptosis is induced by the accumulation of ceramides [196,197]. Thus, ceramides and sphingosine can promote inflammation by increasing apoptosis and cytokine production, while S1P has opposing effects.

Immune cell infiltration and migration into inflammatory sites, as well as their function, are dependent on S1P, ceramide-1-phosphate, and the hydrolysis of sphingolipids [193,198,199]. S1P induces the migration of dendritic cells and the differentiation of monocytes into dendritic cells [200,201,202,203]. S1P can promote the M2 macrophage phenotype and its recruitment to atherosclerotic plaques [204,205]. Sphingolipids also target the adaptive immune system by regulating memory T cells. Increased S1P concentrations recruit lymphocytes with an increased expression of its receptor, S1P receptor 1 (S1PR1), into the lymphatic ducts [206]. In addition, the downregulation of S1PR1 helps to maintain memory T cells in non-lymphoid organs [207]. Signaling via this receptor directly impacts T-cell homing and the differentiation of memory T cells [208]. Lastly, Kleinwort et al. discovered that S1P differentially affects the migration of peritoneal B cells in vitro [209]. However, there is very little evidence on the effect of sphingolipids and B-cell functions.

Sphingolipids are present throughout the GI tract but are more abundant in IECs of the small intestine than in the colonic mucosa [210]. Sphingolipids affect gut barrier function by promoting the proliferation and tight junction formation of IECs [211]. However, ceramide can promote inflammation via NF-κB in IECs but may also be necessary for the formation of the TLR complex in lipid rafts [212]. Ceramide can promote IL-8 production and trigger inflammation by acting as a ligand for TLR4 [213,214]. Lastly, ceramide can decrease barrier function in IECs [215,216]. Interestingly, a higher ratio of fecal sphingolipids (sphingomyelin, ceramide, S1P, and ceramide-1-phosphate) to cerebrosides and gangliosides was found in dextran sodium sulfate (DSS)-treated mice [217]. Sphingomyelin induced the apoptosis of IECs via its hydrolysis to ceramide, while phosphatidylcholine mitigated these effects in vitro [215,218]. Lastly, sphingomyelin consumption in DSS-treated mice reduced colon inflammation and immune cell infiltration, and it improved recovery but augmented chemokine receptor interactions and helper T-cell maturation gene expression [219]. These disparities in the effects of sphingolipids on colitis could be due to potential dietary lipid interactions that may result in alterations in sphingolipid metabolism and may affect the functional properties of sphingolipids. In line with this, dietary fat content can alter colitis and influence the anti-inflammatory effect of milk polar lipids, a source of dietary sphingolipids. Mice fed milk with added sphingolipids were protected against colitis when fed a high-fat diet, while administering milk with added sphingolipids during a low-fat diet attenuated disease activity during the colitis induction period yet promoted colitis and inflammation in male mice during the recovery period [220].

**Figure 3:**
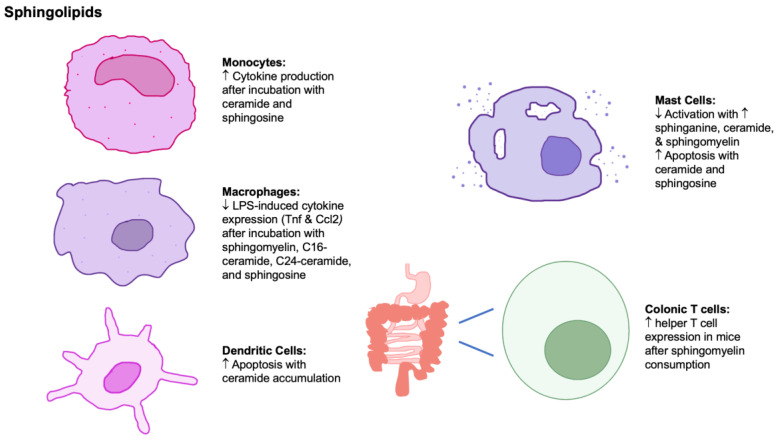
The effects of sphingolipids on innate, adaptive, and mucosal immune cells. Abbreviations: ↑, increase; ↓, decrease; Ccl2, chemokine ligand 2; LPS, lipopolysaccharide; Tnf, tumor necrosis factor.

Overall, the effects of sphingolipids on various immune cells are context-dependent, and they have been shown to affect monocytes, macrophages, dendritic cells, mast cells, and colonic T cells (Figure 3). Further research is required to understand the effects of dietary sphingolipids on the immune cell response under different dietary conditions.

## 4. Factors Altering Lipid–Immune Interactions

Sex plays a role in the differences between lipid metabolism and the immune response. For example, there are differences in the inflammatory response against adipose lipolysis in females and males. In response to stimulated lipolysis, lipolytic activity is enhanced in females with higher levels of serum FFA, IL-6, and triglycerides, which was associated with increased crown-like structures and MCP-1 expression [221]. In men, diacylglycerol, ceramides, phospholipids, and some fatty acid species were correlated with inflammation in gonadal adipose [221]. In addition, estrogen can regulate lipid metabolism and provide protection against cardiovascular disease [222]. Men are prone to produce more triglyceride-rich very-low-density lipoprotein (VLDL) particles, while women also produce triglyceride-rich VLDL but have an increased clearance of these particles [223,224]. There are also differences in inflammatory signaling and immune cell counts. Based on in vitro, in vivo, and ex vivo experiments, females have been found to have increased TLR pathway expression and higher TLR7 expression, while TLR4 signaling in macrophages and neutrophils is higher in males, with an increased production of proinflammatory cytokines [225,226,227,228]. Since TLR expression differs between sexes, with TLR3, 7, and 9 being expressed higher in females and TLR2 and TLR4 in males, the strength of TLR-dependent responses is also biased towards sex differences [229]. In female mice, neutrophil phagocytic capacity and macrophage activation and phagocytic capacity are increased [230,231]. Of the adaptive immune cells in healthy adults, CD4^+^ T-cell counts are increased in females, while males have higher levels of cytotoxic T cells, yet the activity of cytotoxic T cells is increased in females [232,233,234,235,236]. Healthy females also have an increased activation and proliferation of T cells and B cells, with a subsequent increase in antibody production from B cells, while males have increased regulatory T-cell counts [232,237,238,239,240]. Lastly, treatment dosage and genetic differences may contribute to the sex bias in immune responses and the female predominance of autoimmune diseases [229].

Various environmental factors, including lifestyle and diet, can alter the composition of the gut microbiome; this, in turn, can affect metabolic processes and inflammation, which can impact each other [241]. The presence of the gut microbiome stimulates the differentiation of RORγt + NKp46 + NK-like cells and mitigates the proliferation of invariant NK T cells, while segmented filamentous bacteria prompt the differentiation of Th17 cells [242,243,244,245]. Specific microbial populations, such as *Bacteroides fragilis, Clostridia IV*, *and Clostridia XIVa*, can induce Treg differentiation in circulation and the lamina propria, which can mitigate Th17 inflammation by increasing IL-10 secretion [246,247]. On the extreme ends of the inflammatory response, the gut microbiome can impact hyper-immunity, the overexpression of proinflammatory cytokines, and immunodeficiency from genetic mutations in regulatory immune proteins [241]. In contrast, lipid metabolism and microbial populations are tightly intertwined, as lipids can affect bacterial populations, which can then impact the immune response. It has been proposed that the consumption of a HFD can impair the survival of *Bacteroidetes*, which produce lipids that have been shown to have anti-inflammatory properties [248].

There is contrasting evidence on how the microbiome impacts lipid metabolism. Velagapudi et al. investigated the differences in lipid metabolism between germ-free and conventional mice and found increased lipid clearance with decreases in serum triglyceride levels and increased adipose and hepatic triglycerides in the presence of the gut microbiome [249]. Germ-free mice fed a HFD (60% kcal from fat) for 11 weeks were resistant to increases in serum cholesterol and saw increases in fecal cholesterol excretion [250]. There was also an upregulation of hepatic cholesterol biosynthesis; HMG-CoA reductase (*Hmgcr*); and sterol transporter genes, ATP binding cassette subfamily G members 5 and 8 (*Abcg5* and *Abcg8*) [250]. Thus, the gut microbiome is a key factor that can alter many body processes and functions, including lipid metabolism and immune responses.

Many chronic metabolic diseases, such as obesity and T2DM, are also characterized by low-grade inflammation and disturbances in lipid metabolism [251]. Obesity is a chronic disease resulting from the excess storage of fat in adipose tissue that can lead to atypical storage in other organs, such as the liver and muscle, inducing insulin resistance. Insulin resistance occurs due to lipotoxicity and inflammation causing fat deposition into tissues but also promotes inflammatory signaling, which can lead to T2DM. During obesity, macrophages within adipose tissue secrete proinflammatory cytokines, like TNFα, and release FFA, which causes insulin resistance [252]. Individuals with T2DM, insulin resistance, and obesity also have dyslipidemia characterized by increased triglycerides, small dense low-density lipoprotein particles, and reduced HDL-C [253,254,255]. HDL-C concentrations decrease during inflammation via increased HDL catabolism and decreased cholesterol efflux capacity in addition to decreased cholesterol efflux from cells [256]. TNFα and LPS can activate TLRs that inhibit liver X receptor alpha (LXRα) expression and reduce ATP binding cassette subfamily A member 1 (ABCA1), a lipid transporter protein responsible for cholesterol efflux, subsequently decreasing ABCA1-mediated HDL biogenesis and apoA1-mediated cholesterol uptake [257,258,259]. In Chinese adults, serum cytokines and thiobarbituric acid reactive substances (TBARSs), a marker of lipid peroxidation, were positively associated with obesity and T2DM [260]. In adults with heart disease, inflammatory markers were negatively associated with HDL-C and positively associated with triglycerides and LDL-C [261,262,263]. In conclusion, there are disruptions in the interactions between inflammation and lipid metabolism in individuals with compromised metabolic status.

Sphingolipid metabolism is altered during metabolic disease, which can impact the immune function of cells. During obesity, de novo ceramide synthesis increases during inflammation and insulin resistance due to an influx of FFA increasing a ceramide precursor, palmitoyl CoA, and by activating TLR4 on immune cells [264]. The activation of TLR4 increases acid sphingomyelinase activity increasing cellular ceramide concentrations [212,265]. It was found that an increase in ceramide is required for TLR4-dependent insulin resistance in an obese mouse model, and with parallel decreases in sphingosine-1-phosphate, this has been shown to increase apoptosis [266,267]. Sphingolipid content in the plasma membrane has also been shown to be altered during inflammatory states with increased ceramide content [265]. Ceramide modifies the structure of lipid rafts promoting the formation of receptor and signaling complexes, which are essential for inflammatory signaling [189,268,269,270]. Ceramide-enriched LDL activates TLR4 in monocytes promoting the secretion of proinflammatory cytokines [271]. In contrast, reductions in cholesterol and sphingomyelin in lipid rafts cause impaired T-cell activation attenuating inflammation [272]. Thus, during altered metabolic states, sphingolipid and cholesterol metabolism alter the activation of pattern recognition receptors and immune signaling via their role in lipid rafts.

## 5. Current Gaps in Knowledge and Future Directions

It is clear that lipid metabolism and the activation/suppression of the immune system are interconnected. As a result, these lipid and immune cell interactions are important contributors to the risk and pathogenesis of diseases. Endogenous and dietary lipids can act as signaling molecules altering the growth, differentiation, and metabolism of immune cells. The effect of sphingolipids on immune cell activity should be further studied—specifically, the role of sphingolipids on lymphocyte cell activity and the mucosal immune system, including goblet cells and Paneth cells. However, an issue of many of these studies is that the main model used is an in vitro cell culture, which many times does not directly correlate with what would occur in humans. It would be ideal to isolate, culture, and treat cells from human blood and/or tissue samples, which would provide more accurate information about how immune cells behave. In addition, researchers should consider sex effects since there are observed differences in lipid metabolism and immune response between males and females. Other factors such as disease models and genetics play a confounding role and should also be considered.

Future studies need to account for the lipid source, duration, and base fat content of the diet when identifying the properties of bioactive lipids. These differences can affect how lipids interact with immune cells and promote or suppress disease states. Comparisons should be made between different sources and compositions of bioactive lipids to determine which has stronger bioactive properties. Furthermore, nutrition studies should consider that foods are eaten in concert and that the food matrix is critical for determining functional properties. Lastly, the gut microbiome is an abundant source of metabolites that play a major role in host health, including bioactive lipids, which are directly affected by diet. While there is evidence that bacterial and microbial lipids can interact with the immune system and alter immune responses [273,274], further research needs to be conducted to identify mechanisms, sex-dependent differences, and other factors that alter this relationship.

## Data Availability

Data sharing not applicable.
